# Supplementary figures and images for: PyFibers: An open-source NEURON-Python package to simulate responses of model nerve fibers to electrical stimulation
Source: PLoS Comput Biol. 2025 Dec 12;21(12):e1013764. doi: 10.1371/journal.pcbi.1013764 (PMC12700385; doi:10.1371/journal.pcbi.1013764)

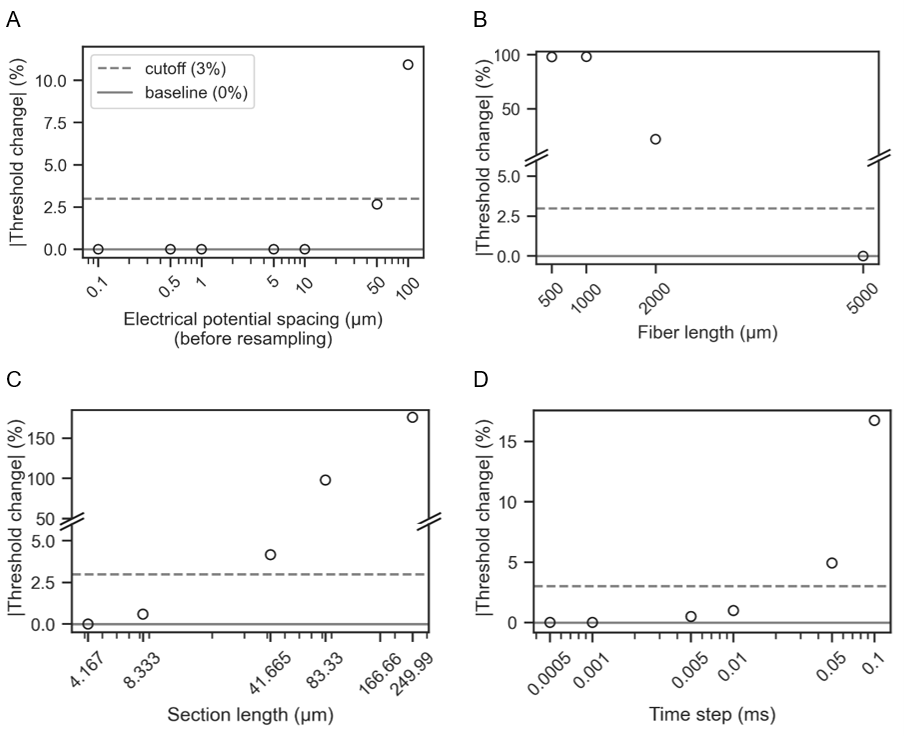

Supplement: S1 Fig — Effects of spatial and temporal parameters on activation thresholds. Stimulation of a 1 μm diameter Tigerholm fiber with a length of 2 mm. The stimulation potentials were from a point current source located halfway along the fiber at an electrode-fiber distance of 100 μm in an isotropic, homogeneous medium with a conductivity of 1 S/m. The stimulation waveform was a monophasic cathodic rectangular pulse with pulse duration of 1 ms at t = 0. The simulation used a time step of 0.005 ms. Parameters were varied from the preceding description as specified in each panel; for each panel, threshold change was calculated from the “best” parameter. A) Distance between consecutive coordinates of electric potentials from a point current source, which were then resampled to match the distance between the centers of the fiber sections (8.333 μm). B) Fiber length. C) Section length. D) Time step. (TIF) [file pcbi.1013764.s001.tif]

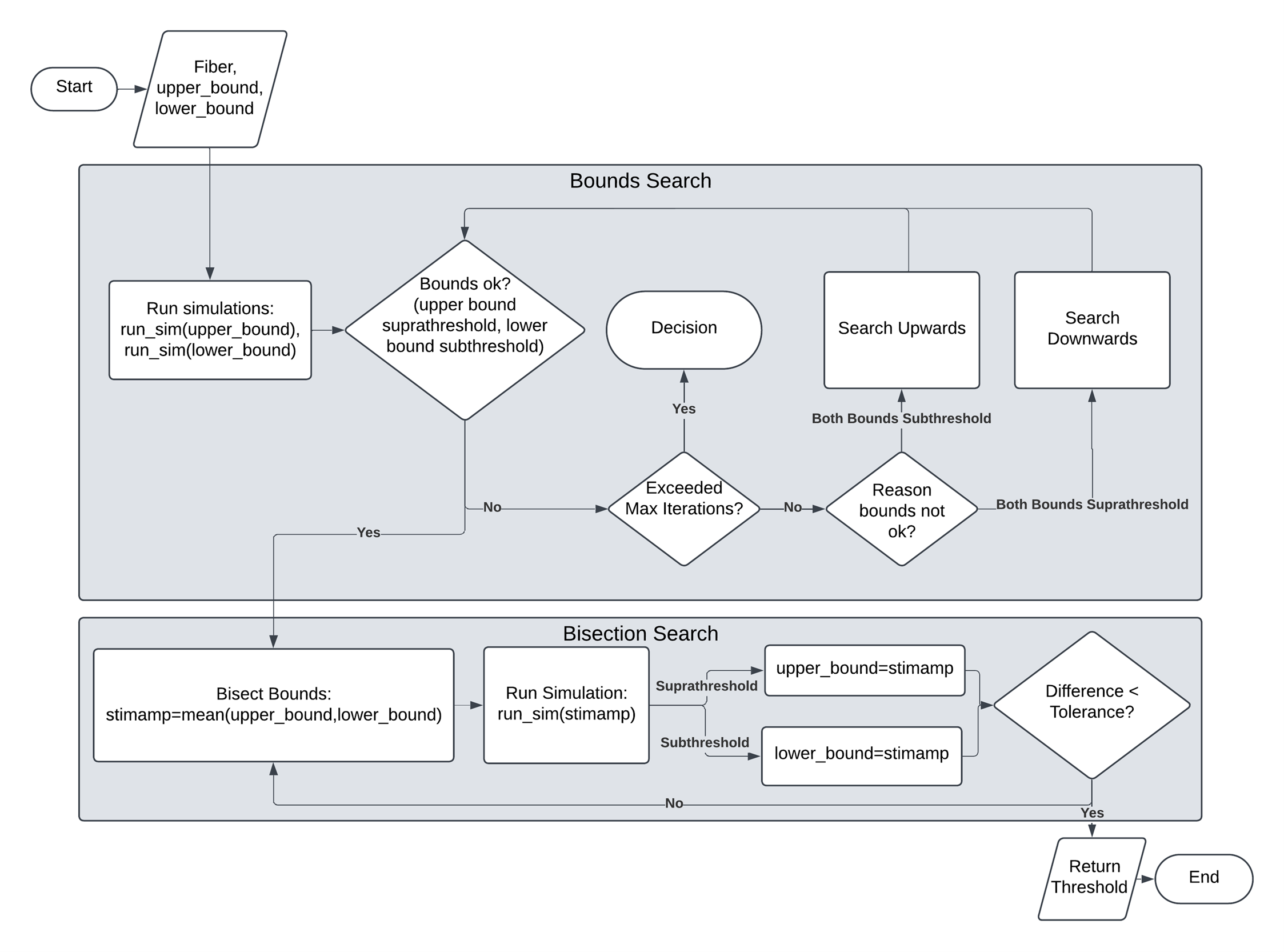

Supplement: S2 Fig — For simplification, several validation checks and details of steps are omitted or simplified. Initial upper and lower bound amplitudes are provided as user inputs. If the bounds are too low (both subthreshold), an upwards bounds search commences, and if the bounds are too high (both suprathreshold), a downwards bounds search commences. Once the bounds are established (lower bound subthreshold, upper bound suprathreshold), a bisection search executes until the user-defined exit criterion is reached. Note: During block threshold searches, the sub/suprathreshold check is delayed until after a user provided “block_delay” argument. If the stimulus generates action potentials after this delay, the stimulus will be considered subthreshold. (TIF) [file pcbi.1013764.s002.tif]

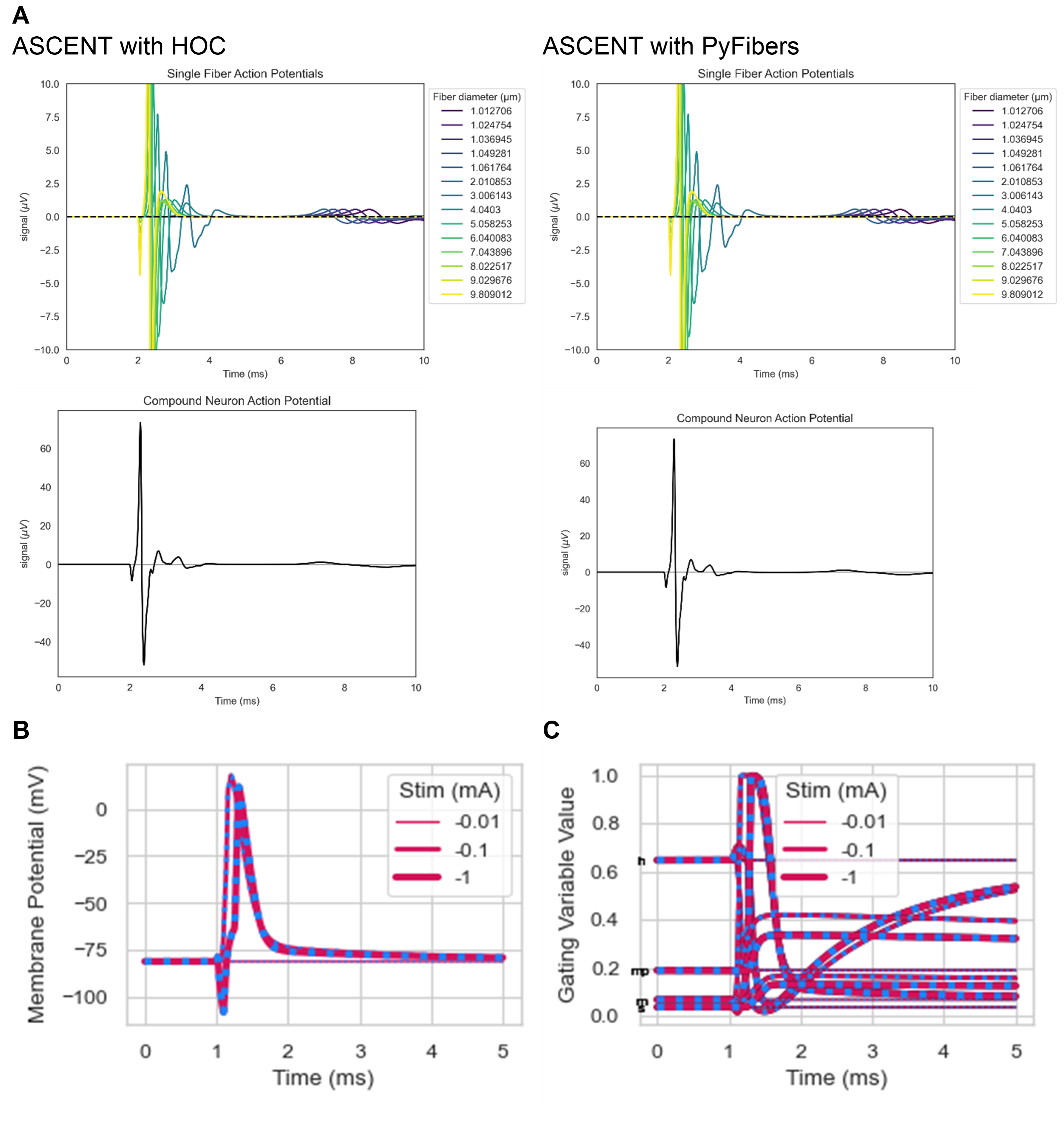

Supplement: S3 Fig — A) Comparison of single fiber action potentials and compound action potentials using Peña fibers in ASCENT with HOC (left) and ASCENT with PyFibers (right). Simulation used the ASCENT tutorial files for CAP recording. The bottom row is the sum of SFAPs from the top row. B, C) Comparison of transmembrane potentials (B) and gating variables (C) over time for the center node of a 4 μm diameter Peña fiber in ASCENT with HOC (solid red) and ASCENT with PyFibers (dashed blue), using the model described in “Leveraging PyFibers with ASCENT to simulate activation and recording of a model nerve”. (TIF) [file pcbi.1013764.s003.tif]

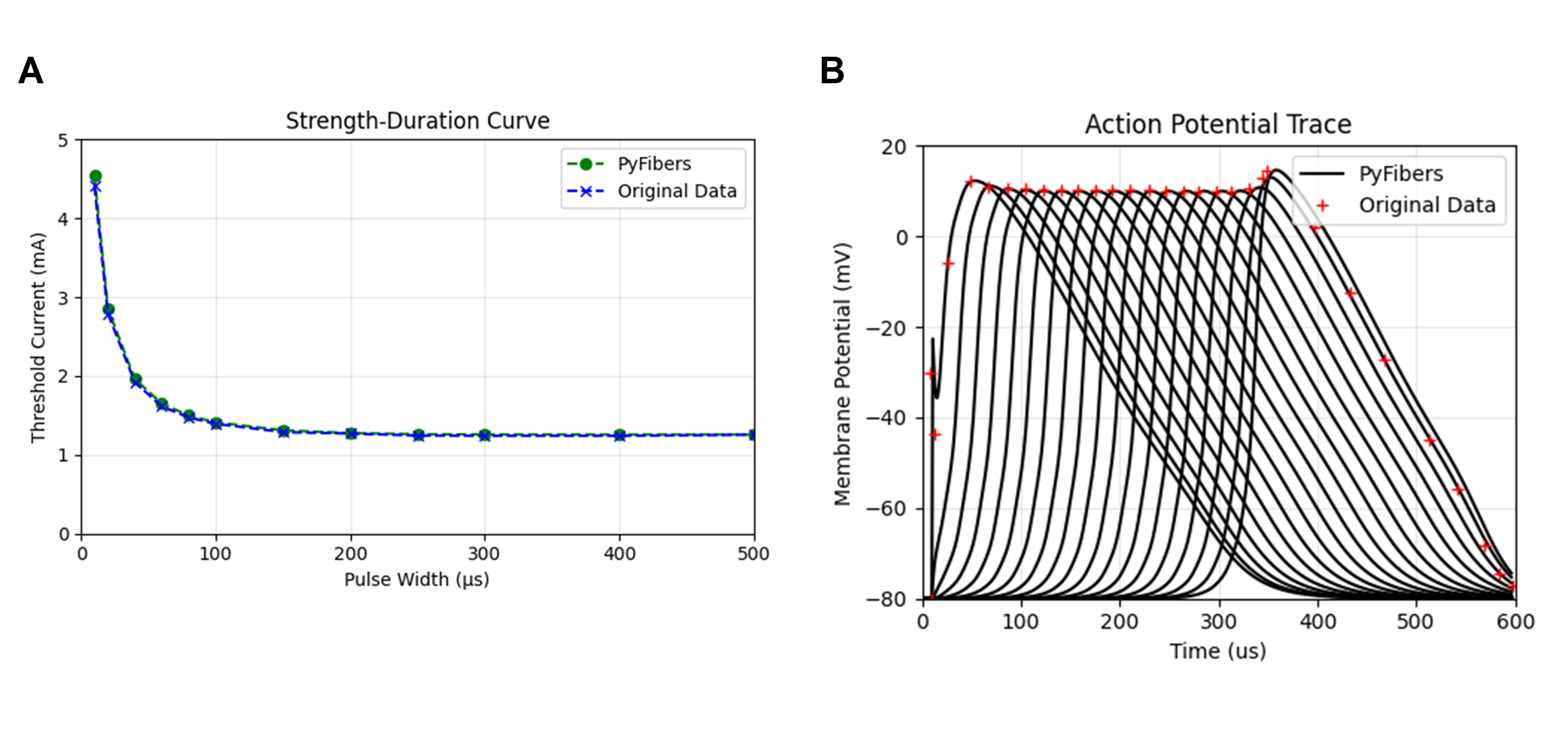

Supplement: S4 Fig — A) Strength-duration curve. B) Action potential conduction. (TIF) [file pcbi.1013764.s004.tif]

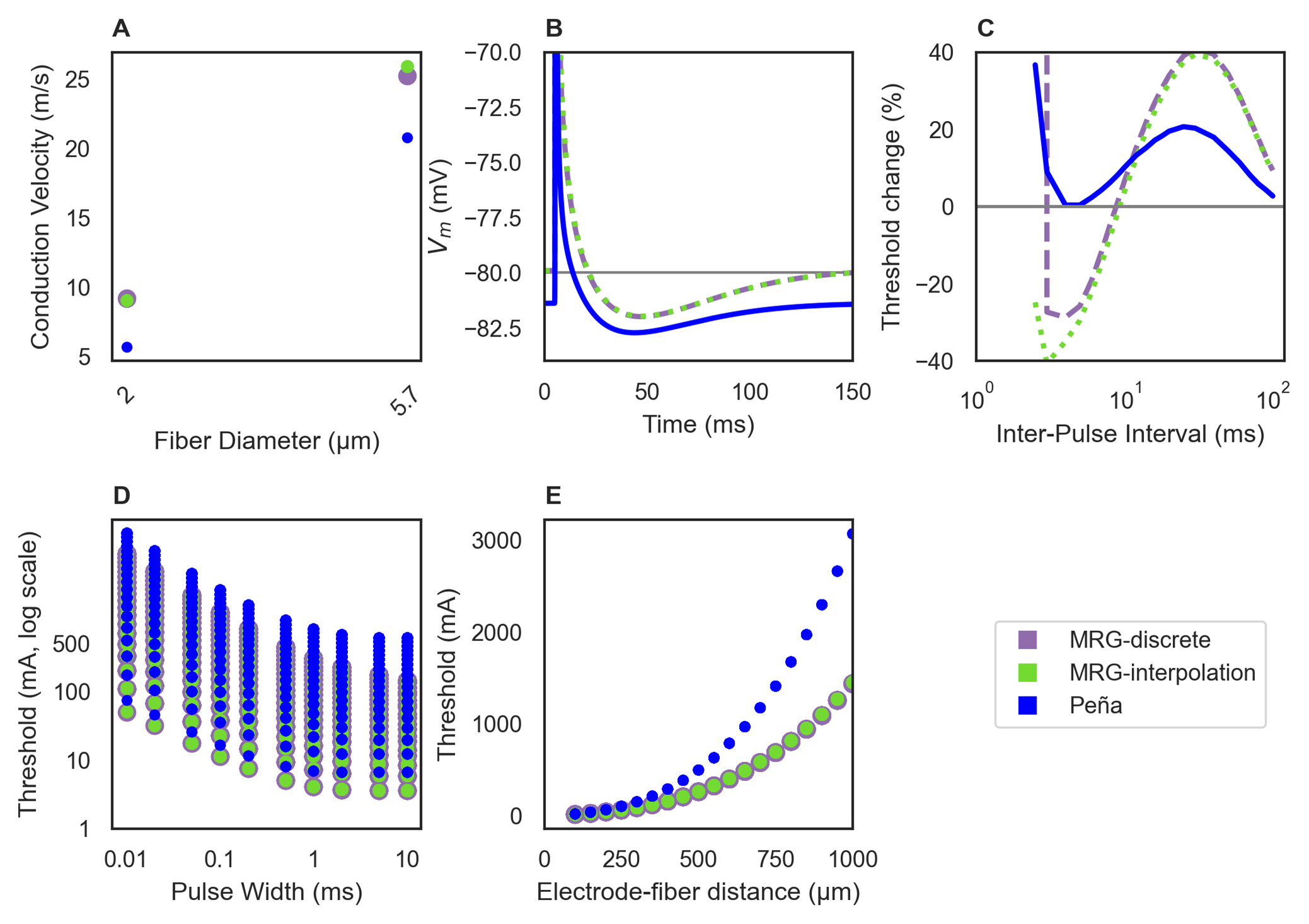

Supplement: S5 Fig — (TIF) [file pcbi.1013764.s005.tif]

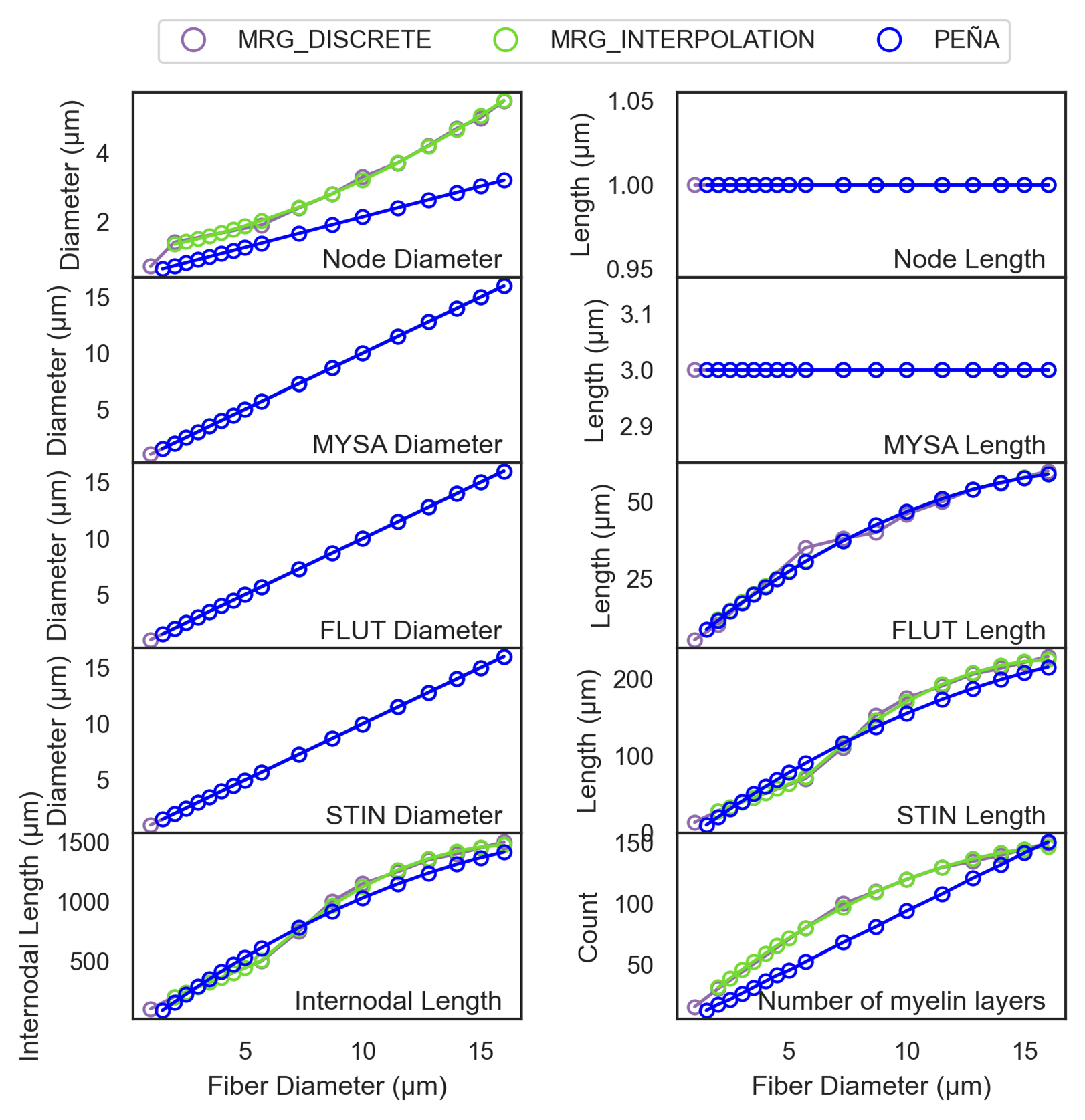

Supplement: S6 Fig — (TIF) [file pcbi.1013764.s006.tif]

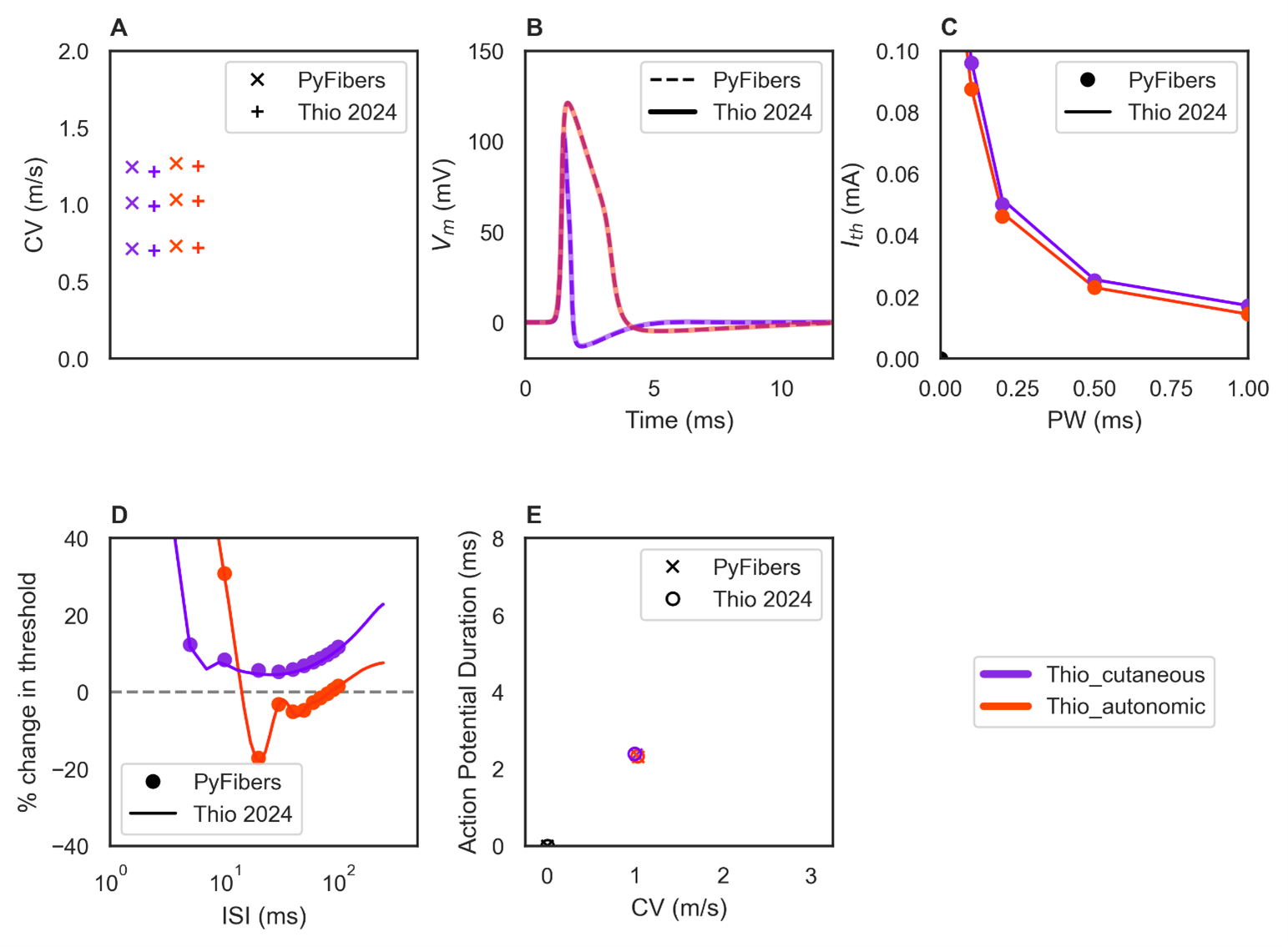

Supplement: S7 Fig — Comparison of Thio fiber model outputs in PyFibers versus published data, confirming that conduction velocity, action potential shape, strength-duration curve, recovery cycle, and action potential duration match previously reported values. We used the same methods as reported in the original publication, including simulating all fibers at 37°C, except for panels C and D, where the Thio cutaneous fiber was simulated at 33°C, and panel E, where action potential duration was measured at 24°C. Except for panel A, all simulations were 1 μm diameter fibers. A) Conduction velocity for 0.5, 1, and 1.5 diameter fibers. B) Action potential shape. C) Strength duration relationship. D) Recovery cycle. E) Conduction velocity vs action potential duration. (TIF) [file pcbi.1013764.s007.tif]
