# Supplementary material for: PyFibers: An open-source NEURON-Python package to simulate responses of model nerve fibers to electrical stimulation
Source: PLoS Comput Biol. 2025 Dec 12;21(12):e1013764. doi: 10.1371/journal.pcbi.1013764 (PMC12700385; doi:10.1371/journal.pcbi.1013764)
Supplement: S1 Text — (DOCX) [file pcbi.1013764.s008.docx]

If users are modeling axons of passage, they must ensure that their model fiber is long enough to avoid action potential initiation at the end of the fiber (i.e., “end excitation”) (Fig 1A), which is not physiologically accurate if the model is intended to represent a nerve that continues beyond the modeled length. Fibers are activated proportionally to the *second* spatial difference extracellular potentials, which typically peaks at the location of the stimulating electrode (Rattay, 1998; Rubinstein, 1993). However, the “sealed ends” of model fibers cause the driving force for transmembrane current flow to be proportional to the *first* spatial difference of extracellular potentials at the fiber end. For fibers that are too short, the driving force at the end nodes can be greater than under the stimulus, and thus result in end excitation (Fig 1A). Therefore, the ends of the fiber must be sufficiently distant from the electrode (Fig 1B) such that the first difference of extracellular potentials at the ends is reduced relative to the second-difference-based driving force at the stimulus (Fig 1C - 1D). The likelihood of end excitation can be further reduced by excluding non-linear voltage-gated ion channels from end nodes (i.e., by using “passive” nodes); by default, PyFibers models each end node of a fiber with passive properties. PyFibers automatically checks for end excitation (Fig 1A); detection of end excitation raises a warning by default, but the user can instead choose to throw an error to terminate the simulation.


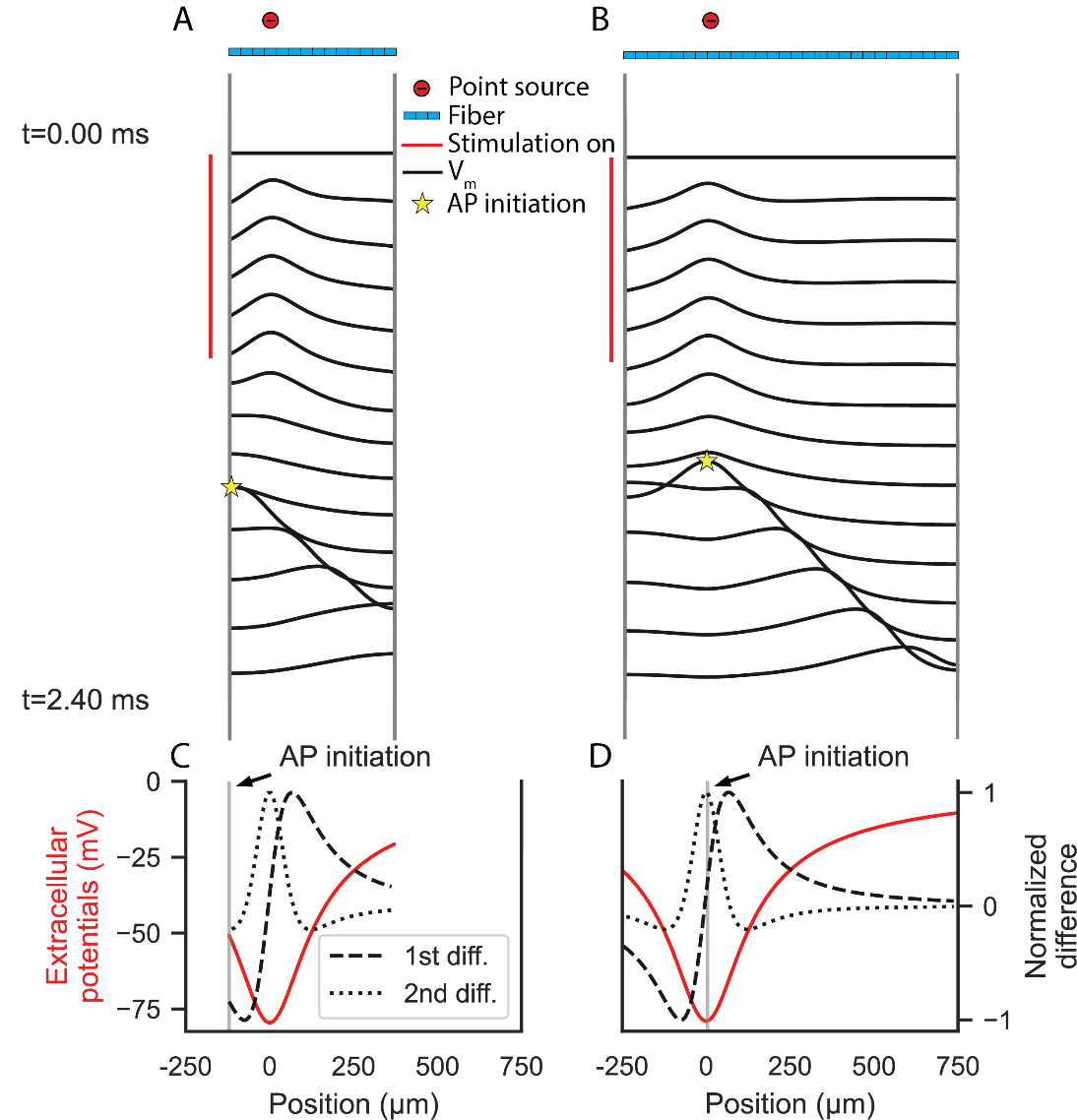


Fig 1: Identifying and resolving end excitation. Stimulation of a 1 μm diameter Tigerholm fiber. The stimulation potentials were from a point current source located halfway along the fiber at an electrode-fiber distance of 100 μm in an isotropic, homogenous medium with a conductivity of 1 S/m. The stimulation waveform was a monophasic cathodic rectangular pulse with pulse duration of 1 ms at t = 0. First and second differences of the transmembrane potential (V_m_) along the fiber were calculated from the electrical potentials sampled at the center of each node, and then normalized to max value of 1 (right y-axis on panels C and D). End excitation occurs when the source is too close to the end of the fiber **(A)**, driven by the first difference **(C)**. End excitation can be avoided by making the fiber longer **(B),** thereby increasing the distance of the fiber ends from the stimulus and reducing the first difference at the ends of the fiber **(D)**. V_m_ = transmembrane potential, AP = action potential.
