# Supplementary material for: PyFibers: An open-source NEURON-Python package to simulate responses of model nerve fibers to electrical stimulation
Source: PLoS Comput Biol. 2025 Dec 12;21(12):e1013764. doi: 10.1371/journal.pcbi.1013764 (PMC12700385; doi:10.1371/journal.pcbi.1013764)
Supplement: S2 Text — (DOCX) [file pcbi.1013764.s009.docx]

During extracellular stimulation, the following procedure occurs during a call of ScaledStim.run_sim():

For a single source, given the spatial distribution of extracellular potentials at the center of each fiber section:

|  | $V_{e}\left( z \right)\quad\text{for}\quad z=1,2,\ldots,n_{\text{sections}}$ | (1) |
| --- | --- | --- |

and the waveform at each time point:

|  | $W\left( t \right)\quad\text{for}\quad t=1,2,\ldots,n_{\text{timesteps}}$ | (2) |
| --- | --- | --- |

the dot product gives the unscaled extracellular potential at each fiber section and each time point:

|  | $V_{e,\text{unscaled}}\left( z,t \right)=V_{e}\left( z \right)\cdot W\left( t \right)$ | (3) |
| --- | --- | --- |

which can then be scaled by desired stimulation amplitude *a:*

|  | $V_{e,\text{scaled}}\left( z,t \right)=a\cdot V_{e}\left( z,t \right)$ | (4) |
| --- | --- | --- |

Under the principle of linearity, the extracellular potentials from multiple sources (*m*) are summed:

|  | $V_{e,\text{final}}\left( z,t \right)=\sum_{k=1}^{m} a_{k}\cdot\left( V_{k}\left( z \right)\cdot W_{k}\left( t \right) \right)$ | (5) |
| --- | --- | --- |

The final matrix of potentials is applied to each section of the model fiber at each time point.
