# Supplementary material for: PyFibers: An open-source NEURON-Python package to simulate responses of model nerve fibers to electrical stimulation
Source: PLoS Comput Biol. 2025 Dec 12;21(12):e1013764. doi: 10.1371/journal.pcbi.1013764 (PMC12700385; doi:10.1371/journal.pcbi.1013764)
Supplement: S3 Text — (DOCX) [file pcbi.1013764.s010.docx]

***Beta test***

*PyFibers: A Python package for simulating the response of model nerve fibers to electrical stimulation using NEURON*

Daniel P. Marshall, Elie S. Farah, Eric D. Musselman, Nicole A. Pelot, Warren M. Grill

Duke University, Department of Biomedical Engineering

**Thank you** for serving as a beta tester for PyFibers, our Python package designed for modeling electrical stimulation of peripheral nerve fibers. Your willingness to share your time and energy is greatly appreciated; your feedback will be invaluable in refining the software before public release and will be recognized in the acknowledgments.

We outline **4** **specific and 2 open-ended tasks** below. The specific tasks evaluate the core functionalities of PyFibers. If you have the time and interest, your feedback on any other features of PyFibers is more than welcome.

If you run into any roadblocks where **the code throws an error** during installation or execution that you cannot readily debug with the documentation, please email us at [daniel.p.marshall@duke.edu](mailto:daniel.p.marshall@duke.edu) with the details of what you were doing specifically when the error occurred (i.e., steps to reproduce the issue) and the error message (with the full error traceback that is printed to the console). We will help address the issue so that you can proceed with the planned testing.

After completing the tasks, please complete the **feedback form—including the results of your simulations—at the end of this document**. We welcome feedback on the feature set, clarity of the documentation, ease-of-use of the code, and any other aspects of the modeling package.

**Attached files – please do not share in part or in whole:**

- This document (MarshallEtAl_PyFibers_BetaTesting_v5.docx)
- Zipped repository containing code and documentation (pyfibers_main.zip)
- Draft manuscript (Manuscript_pyfibers_v6.2.pdf)
- NEURON .mod file for the Sweeney model (sweeney.mod)
- Original publication describing Sweeney model (sweeney.pdf)
- Outputs of CSV data alongside each plot shown in this document (outputs.zip)

Task 1: Startup and Installation

1. Save and unzip the provided git repository.
2. Follow the installation instructions in README.md.
3. Run example_script.py and confirm that the script runs without errors.
4. The documentation is in docs/build/index.html. After opening in a web browser, you’ll see several relevant sections along the left navigation bar, including:
   1. Tutorials: Useful tutorials on how to run PyFibers
   2. API: Documentation of the various functions and classes in PyFibers
   3. Extending functionality: Contains a section on how to create new fiber models, relevant for the optional task #6 to implement the Sweeny model

Task 2: Fiber Stimulation with Point Source

1. Create a model fiber.
   - Diameter: 4 μm
   - Length: 21 nodes
   - Fiber type: Peña
2. Stimulate the fiber using an extracellular point source electrode:
   - Distance from fiber: 250 μm
   - Distance along fiber: half the fiber length (over the center node of Ranvier)
   - Medium conductivity: 1 S/m (isotropic)
   - Wave type: Single pulse square wave
   - Pulse width: 0.5 ms
   - Simulation duration: 10 ms
   - Stimulus amplitude: -0.5 mA
   - Delay before start of stim: 0 ms
3. Output 2.1: After running the simulation, plot the membrane potential (Vm) over time for the nodes of Ranvier closest to the following locations: [0.5, 0.7, 0.9].
4. Output 2.2: Find the activation threshold.
5. Output 2.3: After the threshold search, plot the membrane potential (Vm) over time in response to the threshold amplitude for the nodes of Ranvier closest to the following locations: [0.5, 0.7, 0.9].
6. Document your results in the feedback form at the end of the document. Compare your results with the provided data (in the feedback form and in the attached CSV).

For reference, here are the stimulation waveform and extracellular potentials:

| 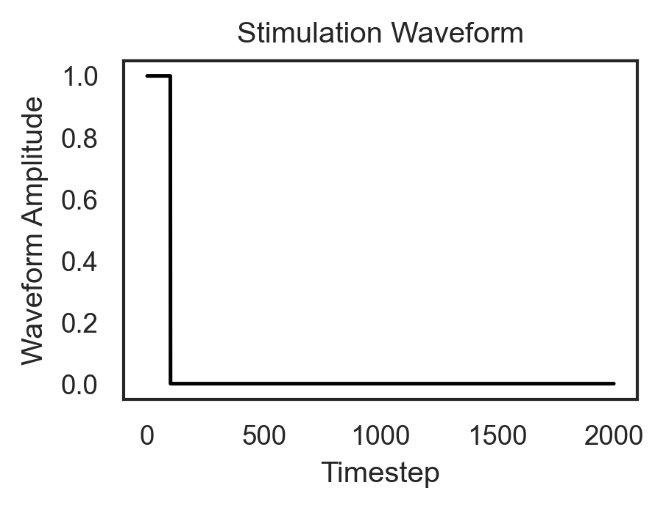 | 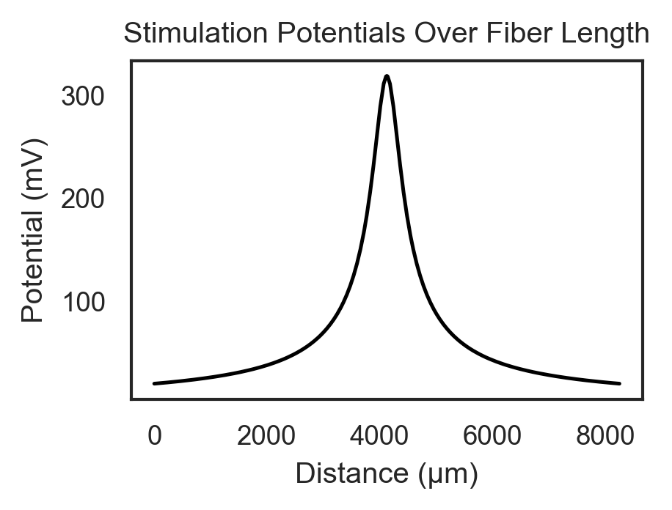 |
| --- | --- |

Task 3: Generate and Plot SFAP

1. Building on Task 2, add a second point electrode to record a single fiber action potential (SFAP) evoked by the extracellular stimulus at threshold amplitude from Task 2.2. Note that this point source is distinct from the point source used to generate extracellular potentials.
   - Location: 10% fiber length, 100 μm from the fiber
   - Medium conductivity: 1 S/m (isotropic)
2. Output 3.1: Plot the SFAP.
3. Document your results in the feedback form at the end of the document. Compare your results with the provided data (in the feedback form and in the attached CSV).

Task 4: Fiber Stimulation with Two Point Sources

1. Building on Task 2, add a second extracellular stimulation point source using the same parameters as the first point source, except:
   - Pulse width: 0.1 ms
   - Distance along fiber: 70% along the fiber
   - Delay before start of stim: 2 ms
2. Set the amplitude of the first source to -0.5 mA and the amplitude of the second source to 2 mA.
3. Output 4.1: Plot the membrane potential (Vm) over time for the nodes of Ranvier closest to the following locations: [0.5, 0.7, 0.9].
4. Document your results in the feedback form at the end of the document. Compare your results with the provided data (in the feedback form and in the attached CSV).

For reference, here are the waveform and extracellular potentials for the second point source:

| 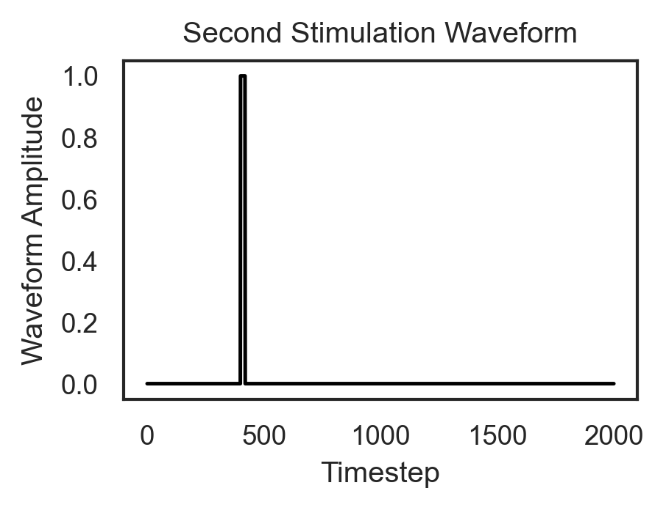 | 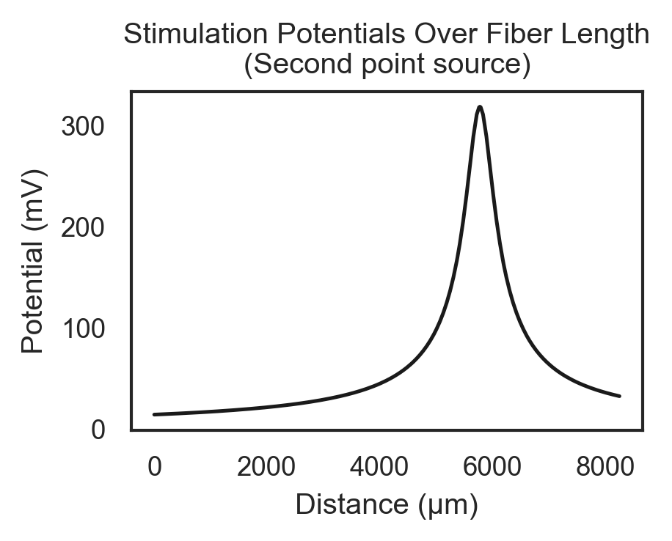 |
| --- | --- |

[Optional] Task 5: Testers’ Choice

We welcome feedback on any other features that you’d like to test. Please let us know:

- what you wanted to test
- whether you were able to complete the test
- what the results were
- any notes on the experience

[Optional] Task 6: Implement a Custom Fiber Model

1. Implement the Sweeney model in PyFibers using the provided sweeney.mod file and PDF of the original publication. NOTE: there are incorrect values in the publication which have been corrected in the provided .mod file: amD =5.3, 𝒈̅𝑳 = 0.128 S/cm2, and 𝒈̅𝑵𝒂 = 1.445 S/cm2 where amD is the highlighted number in the alpham equation, 𝑔̅𝐿 is the leakage conductance, and 𝑔̅𝑁𝑎 is the maximal sodium channel conductance in the “Constants and Relations” section.
2. Create a Sweeney model fiber and calculate the activation threshold using the same setup as Task 2.
3. Output 6.1: Plot the membrane potential (Vm) over time for the nodes of Ranvier closest to the following locations: [0.5, 0.7, 0.9].
4. Output 6.2: Find the activation threshold.
5. Document your results in the feedback form at the end of the document. Compare your results with the provided data (in the feedback form and in the attached CSV).

Feedback

Name

<<answer here>>

Email

<<answer here>>

Affiliation

<<answer here>>

Date

<<answer here>>

Operating system & version

<<answer here>>

Python version

<<answer here>>

NEURON version

<<answer here>>

**Ease-of-use**: Is it clear how to use the package? If not, what aspects are unclear? What points of confusion have you had?

<<answer here>>

**Ease-of-use**: Are there any ways the use of the package could be made easier (through changes in how the package is used/functions/classes)?

<<answer here>>

**Features**: What additional features would you like to see included in PyFibers, in order of priority?

<<answer here>>

**Documentation**: Are there any specific areas where you feel the documentation is lacking or could be expanded?

<<answer here>>

**Documentation**: Any other improvements you would suggest for the documentation?

<<answer here>>

**Issues**: Have you encountered any unresolved bugs or issues? If so, please describe them.

<<answer here>>

Are there any additional resources or support that would be helpful?

<<answer here>>

Do you have any other comments, suggestions, or feedback?

<<answer here>>

Results of tasks:

| Task | Provided Result | Your Results |
| --- | --- | --- |
| Output 2.1 | 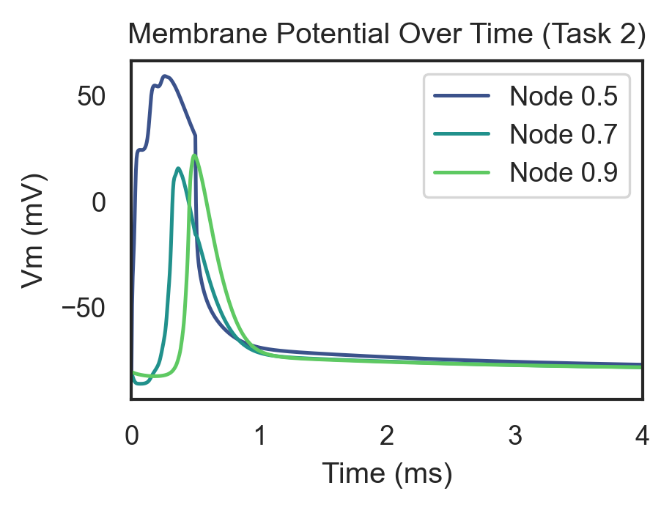 | <<result here>> |
| Output 2.2 | Threshold = -0.08416992187500001 (mA) | <<result here>> |
| Output 2.3 | 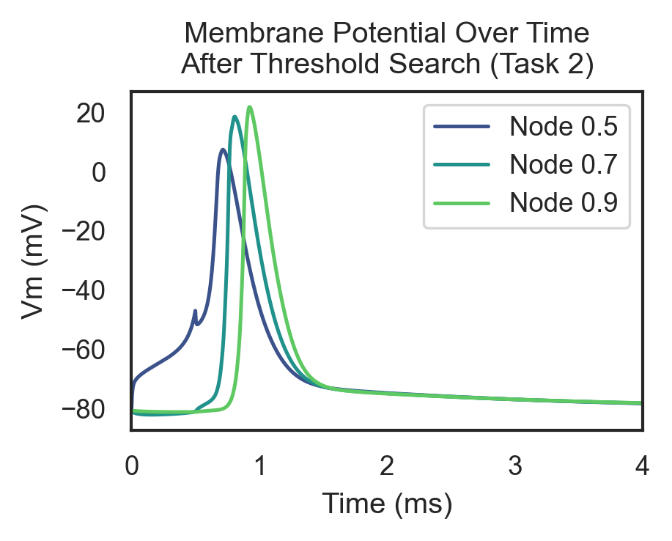 | <<result here>> |
| Output 3.1 | 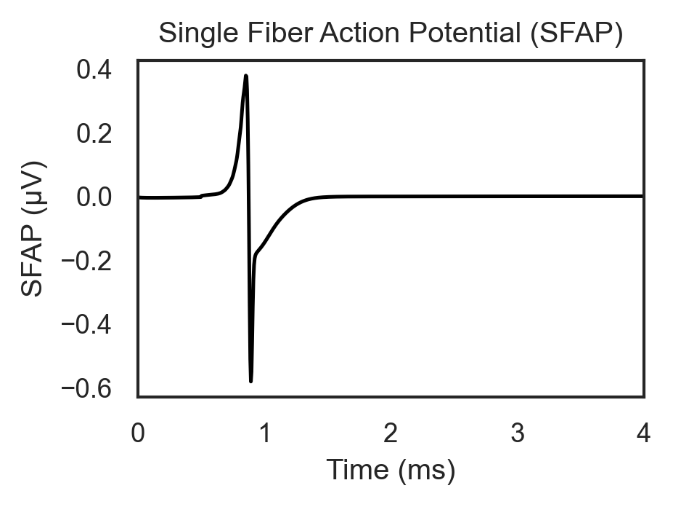 | <<result here>> |
| Output 4.1 | 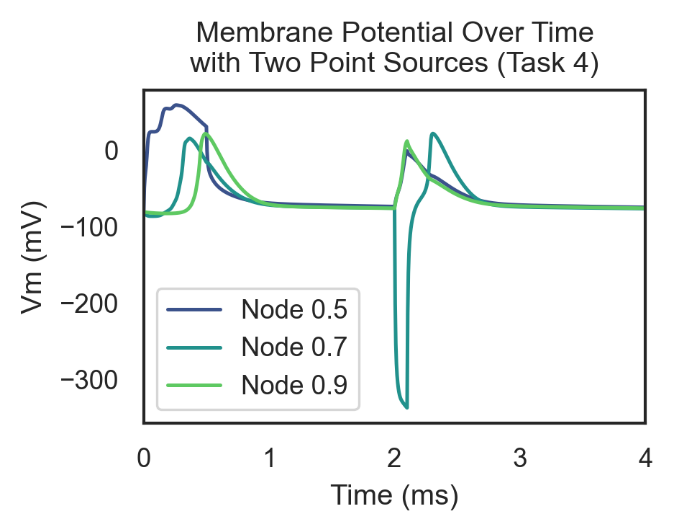 | <<result here>> |
| Output 6.1 | 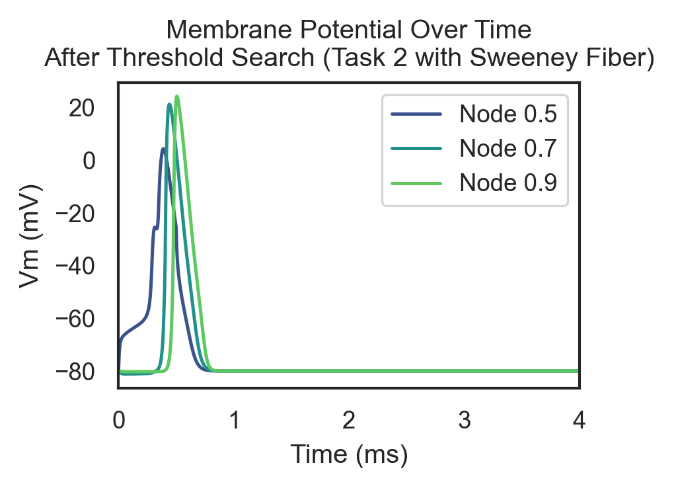 | <<result here>> |
| Output 6.2 | Threshold = -0.11240234375 mA | <<result here>> |
