# Supplementary material for: PyFibers: An open-source NEURON-Python package to simulate responses of model nerve fibers to electrical stimulation
Source: PLoS Comput Biol. 2025 Dec 12;21(12):e1013764. doi: 10.1371/journal.pcbi.1013764 (PMC12700385; doi:10.1371/journal.pcbi.1013764)
Supplement: S4 Text — (DOCX) [file pcbi.1013764.s011.docx]

The code below is the NMODL file associated with the Sweeney example fiber model in the main text. Note that three misprints in the original publication have been corrected in this code: leak conductance = 0.128 S/cm^2^, maximum sodium conductance = 1.445 S/cm^2^, and alpha_m_ constant D = 5.3.

: TITLE sweeney.mod Sweeney Channel

: Sweeney channel fast sodium channel for myelinated axon

: set for a resting potential of -80 mV

NEURON {

    SUFFIX sweeney

    USEION na READ ena WRITE ina

    NONSPECIFIC_CURRENT il

    RANGE gnabar, gl, el, ena, il

    RANGE minf, hinf

    RANGE mtau, htau

    RANGE mexp, hexp

}

UNITS {

    (mA) = (milliamp)

    (mV) = (millivolt)

}

INDEPENDENT {t FROM 0 TO 1 WITH 1 (ms)}

PARAMETER {

    v (mV)

    celsius = 37 (degC)

    dt (ms)

    gnabar = 1.445 (mho/cm2)

    gl = 0.128 (mho/cm2)

    el = -80.01 (mV)

    ena = 35.64 (mV)

    vtraub = 0 (mV)

    amA = 49

    amB = 126

    amC = 0.363

    amD = 5.3

    bmA = 56.2

    bmB = 4.17

    ahA = 56

    ahB = 15.6

    bhA = 10

    bhB = 74.5

    bhC = 5

}

STATE {

    m

    h

}

ASSIGNED {

    ina (mA/cm2)

    il (mA/cm2)

    minf

    hinf

    mtau (ms)

    htau (ms)

    mexp

    hexp

}

BREAKPOINT {

    SOLVE states

    ina = gnabar * m * m * h * (v - ena)

    il = gl * (v - el)

}

: DERIVATIVE states {

:   rates(v) METHOD cnexp

:   m' = (minf-m)/mtau

:   h' = (hinf-h)/htau

:}

PROCEDURE states() {    : exact when v held constant

    rates(v)

    m = m + mexp * (minf - m)

    h = h + hexp * (hinf - h)

    VERBATIM

    return 0;

    ENDVERBATIM

}

UNITSOFF

INITIAL {

    rates(v)

    m = minf

    h = hinf

}

PROCEDURE rates(v) {

    LOCAL v2, alpha, beta, sum

    v2 = v-vtraub

    : m sodium activation system

    alpha = (amB+amC*v2)/(1+Exp(-(amA+v2)/amD))

    beta = (1/Exp((v2+bmA)/bmB))*(amB+amC*v2)/(1+Exp(-(amA+v2)/amD))

    sum = alpha + beta

    mtau = 1/sum

    minf = alpha/sum

    : h sodium inactivation system

    alpha = (1/Exp((v2+bhB)/bhC))*ahB/(1+Exp(-(v2+ahA)/bhA))

    beta = ahB/(1+Exp(-(v2+ahA)/bhA))

    sum = alpha + beta

    htau = 1/sum

    hinf = alpha/sum

    mexp = 1 - Exp(-dt/mtau)

    hexp = 1 - Exp(-dt/htau)

}

FUNCTION Exp(x) {

    if (x<-100) {

        Exp = 0

    }else{

        Exp = exp(x)

    }

}

UNITSON
