# Supplementary material for: PyFibers: An open-source NEURON-Python package to simulate responses of model nerve fibers to electrical stimulation
Source: PLoS Comput Biol. 2025 Dec 12;21(12):e1013764. doi: 10.1371/journal.pcbi.1013764 (PMC12700385; doi:10.1371/journal.pcbi.1013764)
Supplement: S5 Text — (DOCX) [file pcbi.1013764.s012.docx]

We made three changes to the bisection search algorithm for thresholds in PyFibers that dramatically reduced simulation time compared to the HOC implementation in ASCENT.

1. During the initial search for upper and lower bounds, if both bounds are suprathreshold, the lower bound is decreased, and the old lower bound becomes the new upper bound, rather than the upper bound remaining static. The reverse is true when both bounds are subthreshold, with each new increase of the upper bound resulting in its old value supplanting the lower bound.
2. For activation threshold searches only, the simulation immediately exits if an action potential is detected on the “detection node” (by default, the node at 90% fiber length), rather than completing the simulation to the specified tstop.
3. Once (2) occurs for the first time, all subsequent simulations exit at this time plus a buffer (by default, 5 ms).
