# Supplementary material for: PyFibers: An open-source NEURON-Python package to simulate responses of model nerve fibers to electrical stimulation
Source: PLoS Comput Biol. 2025 Dec 12;21(12):e1013764. doi: 10.1371/journal.pcbi.1013764 (PMC12700385; doi:10.1371/journal.pcbi.1013764)
Supplement: Attachment — Submitted filename: PCOMPBIOL-D-25-00791_RESPONSE_v3.docx [file pcbi.1013764.s014.docx]

**Response to Reviewer Comments**

**Submission ID**: PCOMPBIOL-D-25-00791

**Title**: PyFibers: An open-source NEURON-Python package to simulate responses of model nerve fibers to electrical stimulation

# Additional Editor Comments

*The software builds upon libraries of the NEURON suite and innovations ought to be emphasized in the revision. As a "software article" the manuscript could be published as long as the novelty and examples of impact cases not addressed by existing libraries could be stated. In your revision please address additional reviewer comments.*

We thank the Reviewers and Editor for their thorough and constructive feedback. We revised the Introduction to clarify the innovation and novelty of PyFibers and added new Table 1, to clarify the features and advantages of PyFibers relative to other tools, also addressed in response to Q4 from Reviewer 1. We also added a new “Use Cases” section in the Results to make clear the impact of PyFibers, as detailed in response to Q3 from Reviewer 1 and Q2 from Reviewer 2.

# Reviewer 1

*The submission “PyFibers: An Open-Source NEURON-Python Package to Simulate Responses of Model NerveFibers to Electrical Stimulation” describes a well-developed Python package that models and simulates the activity of peripheral nerve fibers within the NEURON simulation environment. It carries the overcoming of several chronic obstacles in the field, such as the NEURON HOC language steep learning curve proprietary barrier, reproducibility issues, and simulation fiber over monolithic non-reusable code.*

*Recommendations for Improvement:*

*Q1: “The mindset of continuously developing the software to address the gaps in usability and standardization is admirable. However, the scientific and methodological novelty does not seem to stand out in this particular case. The principal software engineering achievements that provide the core value are contained within the supporting algorithms and models, which have already been established in earlier literature. Instead, consider rephrasing the framing for the software’s contributions toward positioning it as enabling infrastructure that paves the way for innovation and reproducibility rather than a novel scientific finding.”*

We agree with the Reviewer that PyFibers is an enabling infrastructure to facilitate innovation and reproducibility, and we re-framed the manuscript to focus on these contributions. The Introduction and new Availability & Future Directions section now explicitly describe PyFibers as enabling infrastructure that streamlines simulations of nerve fiber models, including use of established models and algorithms, as well as streamlining the implementation of novel models, thereby improving replicability and reusability.

*Q2: “In my opinion, the manuscript would be improved by including a comparison of performance metrics such as runtime, ease of use (lines of code), and accuracy between PyFibers and the traditional HOC methods. Quantitative benchmarks for various system configurations (e.g., with parallel simulations versus without) could assist potential users in assessing the system’s applicability to their research needs.”*

We agree with the Reviewer and compared runtime, ease of use (lines of code), and accuracy between PyFibers and original HOC implementations. Specifically, we compared runtime for our new activation threshold search algorithm vs. the old implementation with seven fiber models in Figure 11B. We did not include other comparisons of runtime as the Python implementation of NEURON still runs HOC under the hood, and we did not make any other algorithmic changes that would result in reduced runtimes. Rather, the focus of our efforts was the usability of the API. In this vein, we compared lines of code in the Results (Box 2 contains 5 lines of user-defined code, whereas the original HOC implementation had 668 lines of code), and we compared accuracy of multiple stimulation response metrics in Figures 9, 10, and 11A, as well as Supplements 6, 7, and 11. Finally, for demonstration of parallelization, the PyFibers documentation also includes guidance on running parallel simulations: <https://wmglab-duke.github.io/pyfibers/tutorials/6_parallelism.html>.

*Q3: “The manuscript could be enhanced by incorporating more PyFibers use cases in realistic experimental workflows such as incorporating data from electrode arrays and comparing it to electrophysiological recordings. Even a small illustrative example of alignment between PyFibers simulations and actual recordings like SFAPs would demonstrate the utility and impact of PyFibers in simulation.”*

We agree that demonstrations of such enabling technology are important, and we added a new “Use cases” section to the Results. We present applications where PyFibers is used to: (1) simulate activation thresholds from stimulation with a point source, (2) record single fiber action potentials, (3) simulate block thresholds from kilohertz frequency stimulation from a point source, (4) modify properties of the underlying NEURON objects that comprise a model fiber, (5) use ASCENT in concert with PyFibers to model stimulation and compound action potential recording in a model nerve, and (6) implement a new fiber model within the PyFibers framework.

*Q4: “Other tools like ASCENT or those built around HOC are mentioned, but a more thorough discussion would be helpful to identify relative benefits of PyFibers.”*

We agree and revised the Introduction to include specific comparisons of PyFibers with alternatives, summarized in the new Table 1 where we highlight specific features and capabilities of PyFibers that are lacking in other open-source platforms.

*Q5: “Several hyperlinks (to documentation, GitHub, or ReadTheDocs) are listed as placeholders (e.g. <>) which disrupt narrative flow. These should be finalized to ensure reviewers and prospective users will navigate freely throughout the submission.”*

The PyFibers code and documentation are now available, and we replaced all placeholders in the manuscript with live URLs.

- GitHub: <https://github.com/wmglab-duke/pyfibers>
- Docs: <https://wmglab-duke.github.io/pyfibers/>
- PyPi: <https://pypi.org/project/pyfibers/>
- Zenodo: <https://doi.org/10.5281/zenodo.17068760>

*Q6: “The biologically unrealistic parameters such as fiber diameters or absent mechanisms would be drastic user errors for PyFibers. The paper does not talk about how these edge cases would be handled. It would be better with at least minimal mentions of these gaps.”*

PyFibers conducts checks on critical inputs (e.g., fiber diameter) and raises warnings or errors when values fall outside recommended bounds. These bounds are discussed in the revised manuscript (Design and Implementation). Further details on handling of such edge cases in PyFibers are included in our documentation (<https://wmglab-duke.github.io/pyfibers/>). We also emphasize in both the manuscript and the documentation that the user is ultimately responsible to ensure appropriate model design and parameterization.

*Q7: “The choice of fiber models is an important one. Although it is nice to see models from prior work being used, some reasoning behind the rationale for model selection could be discussed. Will there be community-wide mechanisms for model submissions or for extending the model library?”*

We agree with the Reviewer that the choice of fiber model is important, and that is one reason we included a substantial number of validated model implementations in PyFibers. As well, PyFibers is designed to support community contributions to add new fiber models, as described in the new “Availability and Future Directions” section and in the documentation (<https://wmglab-duke.github.io/pyfibers/>). New fiber models may be published in separate repositories that are configured to be recognized as plugins for PyFibers; these fiber models will then be available in PyFibers on installation of the plugin. In addition, in the new “Use Cases” section, we demonstrate the implementation of a new fiber model.

*Q8: “Additional overview schematics, not only based on the simulation flow diagrams, would aid users in understanding the holistic interactions among fiber models, simulation protocols, and the analysis tools and their workflows, thus aiding user comprehension. The visualizations provided are clear and informative. Make sure that high-resolution versions are included in the final submission. Specifically, within figure legends, check for consistency in notation (e.g., Vm, vm, Vm(t)) and ensure uniformity across the manuscript.”*

Overview schematics of PyFibers’ design and use are provided in Figures 1 and 5. We revised our figures and captions to ensure uniformity of notation across the manuscript. We uploaded high-resolution .tif images for the figures in the revised submission, per journal guidelines.

*Overall evaluation: This work makes a valuable and innovative contribution to the field of computational neuromodulation. It needs to be improved based upon the comments above.*

# Reviewer 2

*The authors present an open-source Python software library, "PyFibers", designed to provide an efficient computational framework for simulating peripheral nerve fiber responses to electrical stimulation. The library is built upon the classic HOC-based NEURON environment and introduces a modular, extensible framework that includes a baseline of 10 experimentally validated fiber models. The proposed framework also features stimulation models, a user interface, documentation, tutorials, and unit testing. However, at this stage, the code is not yet publicly available.*

*The manuscript was submitted as a “software article” rather than a “research paper,” which is the appropriate classification for this work, and it has been reviewed accordingly.*

*Overall, I find the framework relevant and potentially impactful for computational neuroscientists who aim to simulate morphologically driven phenomena. The proposed system is well-structured, object-oriented, and cross-platform compatible, with apparent integration into existing pipelines such as ASCENT. The package is accompanied by usage guides and API documentation and includes support for both myelinated and unmyelinated fiber models. Importantly, the authors validated their implementations against previously published models.*

*Areas for Improvement*

*Q1: “Benchmarking. The authors highlight a reduction in the lines of code required (which is reasonable), but they should also consider evaluating their framework in terms of quantitative performance metrics, particularly runtime efficiency. Comparing PyFibers to traditional HOC implementations on complex, large-scale morphological models would help demonstrate its practical usability and scalability.”*

We agree with the Reviewer that runtime efficiency is a critical consideration for computational modeling software. With one exception, we did not make any fundamental changes which would alter runtime of the algorithms and models, as our goal was to make established methods more accessible. We did make changes to improve the efficiency of our threshold search protocol, and we compared runtime between PyFibers and the previous HOC implementations for seven fiber models in Figure11B. PyFibers produced a mean reduction in runtime by ~60%.

*Q2: “Functional Use Cases. A primary weakness of the study is the absence of demonstrations involving realistic, functionally significant simulation scenarios. While the authors validate their models against previously published work, they do not showcase how PyFibers can be applied to novel, morphologically driven investigations. While this limitation is somewhat acceptable given the software-focused nature of the manuscript, a discussion on potential applications would significantly enhance the paper’s impact. For instance, referencing studies such as [https://doi.org/10.1371/journal.pcbi.1009754](https://doi.org/10.1371/journal.pcbi.1009754) and discussing how PyFibers could support similar simulations would enrich the discussion and emphasize the tool’s relevance.”*

We agree with the Reviewer and added a new “Use cases” section to the Results. We present applications where PyFibers is used to: (1) simulate activation thresholds from stimulation with a point source, (2) record single fiber action potentials, (3) simulate block thresholds from kilohertz frequency stimulation from a point source, (4) modify properties of the underlying NEURON objects that comprise a model fiber, (5) use ASCENT in concert with PyFibers to model stimulation and compound action potential recording in a model nerve, and (6) implement a new fiber model within the PyFibers framework.
