# Supplementary material for: PyFibers: An open-source NEURON-Python package to simulate responses of model nerve fibers to electrical stimulation
Source: PLoS Comput Biol. 2025 Dec 12;21(12):e1013764. doi: 10.1371/journal.pcbi.1013764 (PMC12700385; doi:10.1371/journal.pcbi.1013764)
Supplement: Attachment — Submitted filename: PCOMPBIOL-D-25-00791R1_RESPONSE_v2.docx [file pcbi.1013764.s015.docx]

**Response to Reviewer Comments**

**Submission ID**: PCOMPBIOL-D-25-00791R1

**Additional Editor Comments:**

Please address the further suggestions for improvement namely:

- Improve the readability of the Design and Implementation section by making it more succinct and accessible to non-programming readers.

- Briefly explain how future community contributions will be moderated or version-controlled.

- Ensure the consistency in notations (e.g., Vm vs. Vₘ, μm vs. um) throughout the figures and tables.

We addressed these reviewer concerns as outlined below.

**Reviewer #1:**

Compared to the original submission, this revised manuscript is significantly better. The authors have now addressed all the previous criticisms by the reviewers and editors in a concise and clear manner. In the revised version, PyFibers is available as open-source, validated computational code, thus greatly enhancing access, reproducibility, and standardization for simulations involving nerve fibers.

We thank the Reviewer for their positive assessment of our work.

Minor comments:

The Design and Implementation section could be tightened slightly to improve readability, particularly for non-programming readers.

We reviewed the Design and Implementation section and made edits to improve clarity and simplify the text, reflected in the updates to the “Operation” subsection. However, an appropriate level of technical detail in the “Implementation” section is necessary. To improve readability, we added the following note to the Operation subsection, as non-programming readers may wish to skip straight to examples of PyFibers-driven simulations: “Further demonstrations of simulations leveraging PyFibers are demonstrated in the “Use cases” section.”

It would also help to briefly explain how future community contributions will be moderated or version-controlled.

The Availability and Future Directions section describes how users may use and expand on PyFibers’ features. We added a note to this section on the ability to add fiber models as plugins described in the main text of the manuscript: “We included the ability for other research groups to publish fiber models as plugins for PyFibers, fostering community development.”

Lastly, ensure consistency in notation (e.g., Vm vs. Vₘ, μm vs. um) throughout the figures and tables.

We conducted a detailed review of our figures and tables. Figure 8 had two cases where we changed um -> μm. We did not identify any other inconsistencies in notation.
